# Femtosecond and Nanosecond Laser Irradiation to Enhance Antimicrobial Activity of α‑Ag**_2–2*x*_** M**_*x*_** WO**_4_** (M**_*x*_** = Cu**_0.15_** and Zn**_0.16_**) Microcrystals

**DOI:** 10.1021/acsomega.6c03200

**Published:** 2026-08-18

**Authors:** Paula F. S. Pereira, Camila C. de Foggi, Ivo M. Pinatti, Thales R. Machado, Henrique Alves, Cristina Paiva de Sousa, Carlos D. Buendía, Gladys Mínguez-Vega, Juan Andrés, Elson Longo

**Affiliations:** † CDMF, LIEC, 67828 Chemistry Department of the Federal University of São Carlos (UFSCar), P.O. Box 676, São Carlos, São Paulo 13565-905, Brazil; ‡ Department of Conservative Dentistry, Faculty of Dentistry, Federal University of Rio Grande Do Sul (UFRGS), Porto Alegre, Rio Grande Do Sul 90035-003, Brazil; § Department of Chemistry, Federal University of Maranhão (UFMA), Avenida dos Portugueses, 1966, São Luís, Maranhão 65080-805, Brazil; ∥ GNANO − Nanomedicine and Nanotoxicology Group, São Carlos, Institute of Physics, University of São Paulo, São Carlos, São Paulo 13566-590, Brazil; ⊥ Department of Morphology and Pathology (DMP), Graduate Program of Biotechnology (PPGBiotec), Laboratory of Microbiology and Biomolecules (LaMiB), São Carlos 13565-905, Brazil; # GROC-UJI, Institut de Noves Tecnologies de la Imatge (INIT), Universitat Jaume I, 12071 Castell'ó'n, Spain

## Abstract

This study investigates the enhancement of the antimicrobial activity of α-Ag_2–2*x*_M_*x*_WO_4_ microcrystals, where M_*x*_ represents Cu_1.15_ and Zn_1.16_, through femtosecond (fs) and nanosecond (ns) laser irradiation. The research highlights how laser exposure induces significant changes in the structural properties of the samples, as evidenced by the analysis of optical phonon modes, which indicate a loss of long-range ordered crystallinity in the irradiated materials. It was demonstrated that this lattice disorder increases the density of available free carriers, potentially enhancing the bactericidal performance of the materials. Furthermore, the study employs techniques such as transmission electron microscopy (TEM) and Fourier-transform infrared (FT-IR) spectroscopy to elucidate the structural order and cluster distortion due to dopant incorporation and laser treatment. The results reveal the presence of amorphous and nonstoichiometric nanoparticles formed during the laser irradiation process, which contributes to the overall enhancement of antimicrobial activity. This work provides a novel approach to improving the antimicrobial efficacy of α-Ag_2–2*x*_M_*x*_WO_4_ microcrystals, paving the way for future research in laser-modified materials to enhance biological activity.

## Graphical abstract

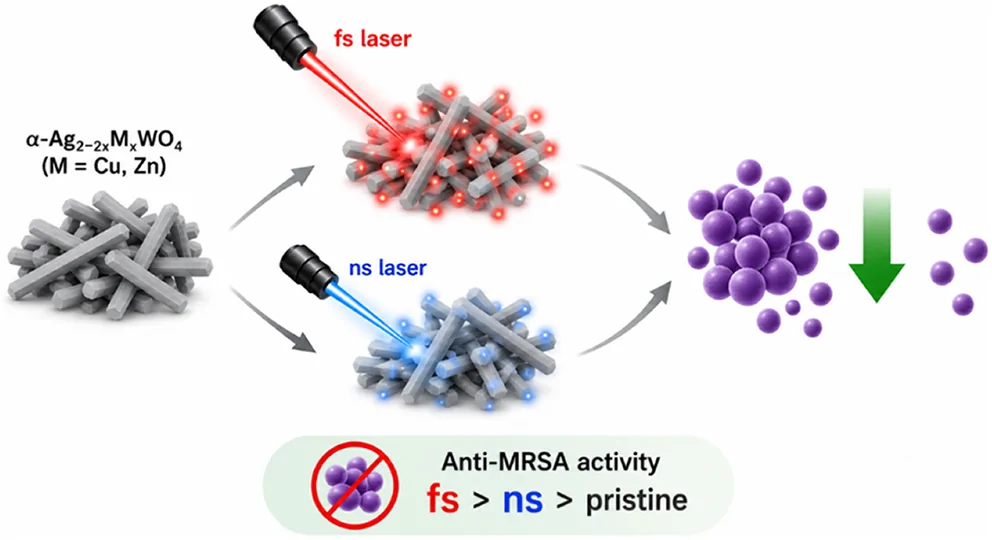

## Introduction

The possibility of tuning the physical and chemical properties of materials with light irradiation opens the door to many different technological applications, like optical sensors or memory devices. Laser irradiation with matter is particularly effective, fast, versatile, clean, and easy to use for the preparation of many types of nanomaterials that cannot be obtained using conventional chemical and physical methods, where the feature size is limited only by the spot size of the laser beam.^1,2^ In this context, laser light with short pulses (femtosecond – fs) or long pulses (nanosecond – ns) is known to cause unique wave–matter interactions with materials and induce the ejection of material composed largely of nanoparticles (NPs) in a broad variety of targets, revealing itself as an amazing tool to produce various NPs and their compounds on the surfaces of several substrates. Therefore, laser irradiation offers a powerful and effective way not only for structural and electronic modifications in materials but also to change material properties and form NPs from bulk structures.^3–5^

α-Ag_2_WO_4_ is an inorganic functional material with potential applications in various fields^6–8^ such as photoluminescence, catalysis, photocatalysis, electrocatalysis,^9–21^ cationic dye removal,^22^ chemical fixation of CO_2_,^23^ lubricants,^24^ and ozone gas sensors.^25^ Moreover, interesting biological properties have been demonstrated for α-Ag_2_WO_4_ and it has been used as an antimicrobial and anticancer agent.^26–43^

In the past, our research group pointed out that the α-Ag_2_WO_4_ irradiated by a femtosecond laser presented a 32-fold improvement in bactericidal properties in relation to α-Ag_2_WO_4_ nonirradiated.^44^ In contrast to the extensively studied pure α-Ag_2_WO_4_, the works devoted to the formation conditions and properties of metal-doped α-Ag_2_WO_4_ are not numerous. In our previous studies, by combining two metals, Ag^+^/ Zn^2+^ and Ag^+^/ Cu^2+^ we obtained the corresponding solid solutions, α-Ag_2–2*x*_Zn_*x*_WO_4_ (0 ≤ *x* ≤ 0.25)^7^ and α-Ag_2–2*x*_Cu_*x*_WO_4_ (0 ≤ *x* ≤ 0.16)^45^ and the properties were enhanced by the synergic effect. To complement these recent experimental works, our research group performed DFT and AIMD simulations to investigate laser-induced changes in the lattice structure and electronic properties of α-, β-, and γ-Ag_2_WO_4_ polymorphs.^46^ Such computational approaches are promising for emerging technologies, particularly when material properties can be tuned by composition, oxygen content, and atomic configurations.

To drastically improve the functionality of the existing α-Ag_2_WO_4_ and/or to develop newly innovative α-Ag_2_WO_4_ material with enhanced properties, it is crucial to understand the electronic behaviors of electrons/holes that can be easily induced by lattice defects, such as substituted impurities and oxygen vacancies. However, these defects simultaneously scatter or trap the doped carriers.^47^ Studies on the effects of laser irradiation on the structural, electrical, and optical properties of these materials have been lacking to date. In the present work, we investigate the effect of nanosecond (ns) and femtosecond (fs) laser irradiation on the structural, morphological, and bactericidal properties of α-Ag_2_WO_4_ and α-Ag_2–2*x*_M_*x*_WO_4_ (M_*x*_ = Cu_0.15_ and Zn_0.16_) samples. The structure was confirmed by X-ray diffraction (XRD), Raman, and Fourier transform infrared (FTIR) spectroscopies. Field-emission scanning electron microscopy (FE-SEM) images were employed to evaluate the shapes and sizes. The morphological analyses of the samples were performed by a high resolution transmission electron microscope (HR-TEM), while the elemental compositions were analyzed by X-ray dispersive energy (EDS) spectroscopy and mapping mode. The presence of metal Ag^+^, as well as Cu^2+^ and Zn^2+^ ions in the α-Ag_2–2*x*_M_*x*_WO_4_ samples, was confirmed by means of X-ray photoelectron spectroscopy (XPS). The antimicrobial activity of the as-synthesized samples against methicillin-resistant *Staphylococcus aureus* (MRSA) was investigated. The present strategy provides an avenue for designing cost-effective and sustainable materials with good performance for technological applications.

## Experimental Section

α-Ag_2–2*x*_M_*x*_WO_4_ (M = Zn^2+^, Cu^2+^; *x* = 0.15, 0.16, respectively) samples were prepared by the CP method, according to our previous work.^7,45^ In a typical experimental procedure, 1 × 10^–3^ mol of tungstate (VI) sodium dihydrate (Na_2_WO_4_·2H_2_O; 99.5% purity, Sigma-Aldrich) and 2 × 10^–3^ mol of silver­(I) nitrate (AgNO_3_; 99.8% purity, Sigma-Aldrich) were dissolved separately in 50 mL of deionized water at 80 °C under magnetic stirring. The solution with Ag^+^ and NO_3_^–^ was added to the solution containing WO_4_^2–^ ions, and this mixture was maintained at 80 °C under magnetic stirring for 30 min. After that, a yellow suspension appeared, and a white precipitate was rapidly formed. The solid solutions were prepared according to the molar ratio in α-Ag_2–2*x*_M_*x*_WO_4_ considering the charge balance between Ag^+^ and M^2+^ (M = Zn^2+^ or Cu^2+^). Zinc nitrate octahydrate (Zn­(NO_3_)_2_·8H_2_O; 99.99% purity, Sigma-Aldrich) and copper nitrate hydrate (Cu­(NO_3_)_2_.*x*H_2_O; 99.99% purity, Sigma-Aldrich) were added separately to the AgNO_3_ solution, and the procedure was similar to that described for the α-Ag_2_WO_4_ synthesis. Finally, the resulting suspensions were washed several times with deionized water to remove the residual Na^+^ and NO_3_^–^ ions. The crystalline α-Ag_2–2*x*_M_*x*_WO_4_ microcrystals were collected and dried in an oven at 70 °C.

### Laser Irradiation

The laser irradiation of pure α-Ag_2_WO_4_ and α-Ag_2–2*x*_M_*x*_WO_4_ (M_*x*_ = Zn_0.15_ and Cu_0.16_) samples was performed with a Ti:sapphire laser (Femtopower Compact Pro, Femto Lasers) emitting pulses of 30 fs full width at half-maximum (fwhm), with a repetition rate of 1 kHz and a central wavelength of 800 nm. Control over pulse duration was obtained by means of an acousto-optic programmable filter (DAZZLER, Faslite) that compensates for dispersion effects and ensures a pulse duration of 30 fs. An iris was used to spatially filter the beam to a diameter of 6 mm at 1/e^2^, and then it was focused onto the surface of the powder target of α-Ag_2–2*x*_M_*x*_WO_4_ (M_*x*_ = Zn_0.15_ and Cu_0.16_) samples using an achromatic 75 mm lens, resulting in a processing pulse energy of 200 μJ with a spot size of 15 μm, a fluence of approximately 113 J/cm^2^ and an energy density of 2260 W/cm^3^. In every tested case, the sample was placed at the bottom of a quartz cuvette attached to a three-dimensional programmable linear stage, which moved in the *x* and *y* directions during irradiation. The radiation pattern depicted in Figure 1 was achieved at a constant velocity of 0.5 mm/s with a mean laser power of 200 mW. The process was repeated three times to ensure homogeneous irradiation of the samples.

**Figure 1:**
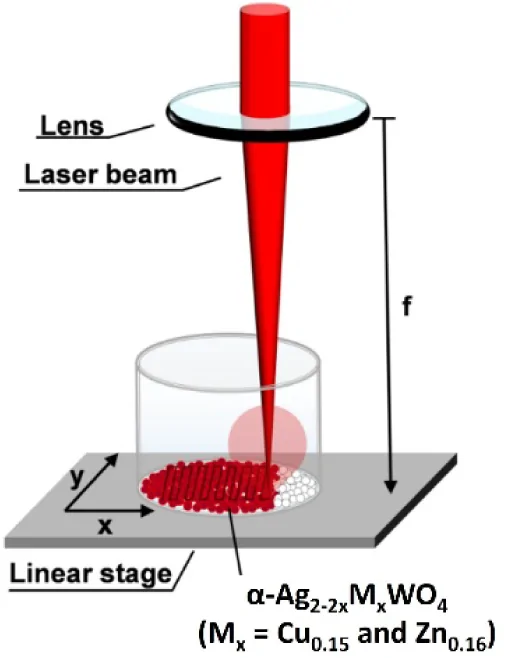
Schematic representation of laser ablation.

To compare the effect of laser irradiation on morphological changes produced in α-Ag_2_WO_4_ and α-Ag_2–2*x*_M_*x*_WO_4_ (M_*x*_ = Zn_0.15_ and Cu_0.16_) samples, irradiation using the second harmonic was performed at 532 nm with a Nd:YAG pulsed laser (Brilliant, Quantel). This source emits pulses of 4 ns (fwhm) at a fundamental wavelength of 1064 nm with a repetition rate of 10 Hz. The laser beam, with an average power of 470 mW, was focused using a 75 mm lens, resulting in a processing pulse energy of 47 mJ with a spot size of 20 μm, a fluence of approximately 15 kJ/cm^2^ and an energy density of approximately 300 kW/cm^3^. The samples were placed in a quartz cuvette attached to a two-dimensional linear stage to perform the same irradiation pattern as in the previous case at a constant velocity of 0.5 mm/s.

### Characterization Methods

The α-Ag_2_WO_4_, α-Ag_1.70_Zn_0.15_WO_4_, and α-Ag_1.68_Cu_0.16_WO_4_ powders, both nonirradiated and irradiated by ns and fs lasers, were characterized by XRD patterns using a D4 Endeavor (Bruker-AXS) with Cu Kα radiation (λ = 1.5406 Å) in the 2θ range from 10° to 70° under normal routine conditions. Raman spectroscopy was conducted on a model NRS-3100 (Jasco) spectrometer equipped with optical microscopy and a CCD detector refrigerated to −65 °C, operating at 613 nm in the range from 250 to 1000 cm^–1^. The spectra were measured in the range of 250–1000 cm^–1^. FTIR spectroscopy was recorded in the range from 400 to 1000 cm^–1^ using KBr pellets as a reference on a model FTIR-6200 (Jasco) spectrophotometer in ATR mode. The shapes and sizes of the α-Ag_2_WO_4_, α-Ag_1.70_Zn_0.15_WO_4_, and α-Ag_1.68_Cu_0.16_WO_4_ nanoparticles were observed with an FE-SEM Inspect F50 (FEI Company, Hillsboro, OR) operated at 5 kV. These nanoparticles were further characterized by HR-TEM using a JEM 2100F TEM/STEM microscope operating at 200 kV and analyzed by EDS. XPS was performed using a ScientaOmicron ESCA+spectrometer with a high-performance hemispheric analyzer (EA 125) and monochromatic Al Kα (hν = 1486.6 eV) radiation as the excitation source. The operating pressure in the ultrahigh vacuum (UHV) chamber during analysis was 2 × 10^–9^ mbar. Energy steps of 50 and 20 eV were used for the survey and high-resolution spectra, respectively.

### Antimicrobial Measurements

The antimicrobial activity of ns and fs, as well as irradiated and nonirradiated α-Ag_2_WO_4_, α-Ag_1.70_Zn_0.15_WO_4_, and α-Ag_1.68_Cu_0.16_WO_4_ samples, was investigated. To assess the antibacterial effect of the synthesized microcrystals, the minimum inhibitory concentration (MIC) and the minimum bactericidal concentration (MBC) against planktonic cells were determined using the assay described by the Clinical and Laboratory Standards Institute (CLSI) with modifications.^48–52^ The MIC was determined as the concentration of the α-Ag_2_WO_4_ solution in which there was no detectable growth, and the CFM value was defined as the lowest concentration that resulted in no growth on the Petri dishes.^53^ A standard strain of Methicillin-resistant *Staphylococcus aureus* (MRSA), obtained from the American Type Culture Collection (ATCC 33591), was used for the test. For inoculum preparation, an aliquot of MRSA was thawed and plated on Mannitol Salt Agar (Acumedia Manufacturers Inc., Baltimore, Maryland, USA) and incubated at 37 °C for 48 h. After incubation, a loop of the microorganism was transferred to 10 mL of Tryptic Soy Broth culture medium (TSB, Acumedia Manufacturers Inc., Baltimore, Maryland, USA) and incubated in an orbital shaker at 75 rpm at 37 °C for 48 h. After this period, the MRSA cells were centrifuged at 5000*g* for 5 min and washed twice with PBS (Phosphate-Buffered Saline, pH 7.2), then suspended again in TSB culture medium. The inoculum concentration was determined using a 600 nm wavelength spectrophotometer, and the optical density (OD) was adjusted to a value corresponding to 1 × 10^7^ cells/mL, for further dilution according to CLSI standard methods. MIC and CBM were determined after incubation of the microorganism in a 96-well culture plate for 24 h at 37 °C, exposed to serial dilutions of the microcrystals in TSB culture medium. A Falcon-type tube containing the culture medium and the microcrystals at the highest concentration (2000 μg/mL) was placed in an ultrasound device for approximately 10 min so that the microcrystals remained suspended in the medium. Then, serial dilutions were performed, obtaining the concentrations of the microcrystals evaluated, which ranged from 1000 to 1.95 μg/mL.^26,31,54^ Culture medium with MRSA and without microcrystals was used as a positive control for the growth of the microorganism, and sterile culture medium was used as a negative control. For both samples, MIC was defined as the lowest concentration of microcrystals that inhibited microorganism growth. For CBM determination, 100 μL aliquots from each well were removed and diluted in microtubes containing 900 μL PBS (10^–1^ to 10^–8^) dilutions. Then, 25 μL of each dilution was plated on plates containing culture medium. The tests were performed in duplicate, and after 48 h of incubation of the plates at 37 °C, colony-forming units per mL (CFU/mL) were calculated, and the result was transformed into log_10_.

All microbiological experiments were performed in triplicate on three different occasions to ensure the reproducibility and accuracy of the data obtained.

## Results and Discussion

### XRD Analysis

The crystalline structures of nonirradiated, fs laser-irradiated, and ns laser irradiated α-Ag_2_WO_4_, α-Ag_1.70_Zn_0.15_WO_4_, and α-Ag_1.68_Cu_0.16_WO_4_ were investigated by XRD analysis, and their corresponding XRD patterns are shown in Figure 2a–i. The Zn and Cu concentrations were chosen considering the maximum crystalline structure capacity without the formation of secondary phases.

**Figure 2:**
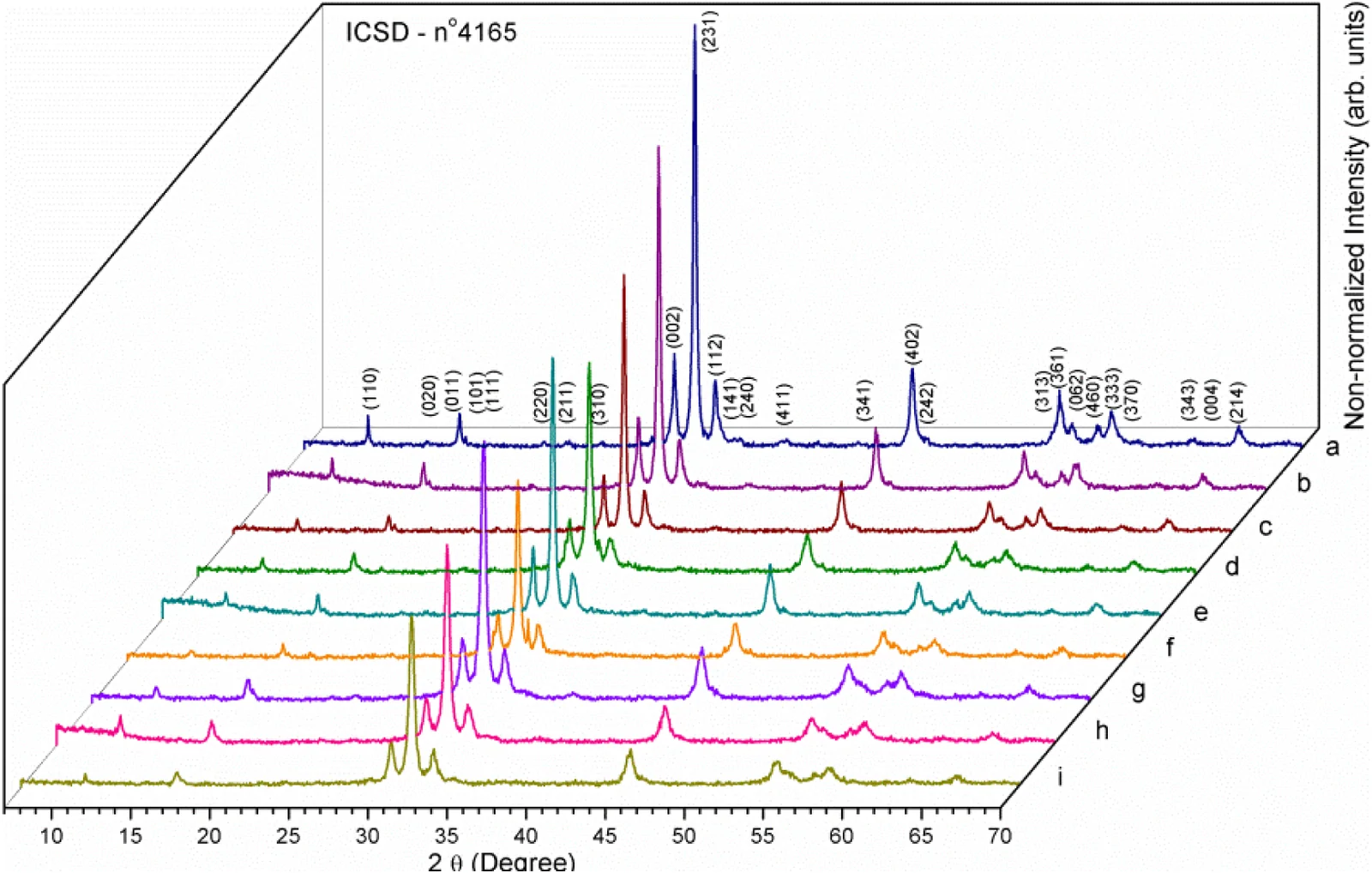
XRD patterns of (a, b, c) α-Ag_2_WO_4_ nonirradiated, ns and fs laser-irradiated; (d, e, f) α-Ag_1.68_Cu_0.16_WO_4_ nonirradiated, ns and fs laser-irradiated; and (g, h, i) α-Ag_1.70_Zn_0.15_WO_4_ nonirradiated, ns and fs laser-irradiated, respectively.

Figure 2a–i illustrates the XRD patterns of all samples, which confirm the orthorhombic structure, space group *Pn2n* and $C_{2v}^{10}$ symmetry. All crystallographic planes are indexed to the Inorganic Crystal Structure Database (ICSD – n^o^ 4165).

Figure 2a, d, and g shows the XRD of nonirradiated α-Ag_2_WO_4_, α-Ag_1.68_Cu_0.16_WO_4_, and α-Ag_1.70_Zn_0.15_WO_4_ samples, respectively. An analysis of the results reveals a decrease in diffraction peaks with the doping process of Zn^2+^ and Cu^2+^ cations in α-Ag_2_WO_4_ crystal lattice. This is a strong indication of the success of the substitution process of Ag^+^ by Zn^2+^ and Cu^2+^ cations, where only a small structural distortion at long range is sensed, as discussed in our previous works.^7^ It is observed for all the irradiated samples that there is a decrease in the intensity of the diffraction peaks and a small shift of the (231) peak, mainly for the samples irradiated by the fs laser. This result can be associated with a larger structural distortion at long range for these samples. In addition, the diffraction peaks of Ag^0^ metallic are not detected, probably due to the lower intensity of the peaks. Similar behavior was observed previously by our research group.^48^

### XPS Spectroscopy Analysis

XPS was used to identify the chemical composition, binding energy, and oxidation state of components present on the material’s surface. The binding energies were obtained by calibrating the spectra using the C 1s peak at 284.50 eV. Figure 3 shows the XPS survey spectra of α-Ag_2–2*x*_M_*x*_WO_4_ (M = Zn^2+^ and Cu^2+^) samples: nonirradiated , ns, and fs laser-irradiated. The main elements identified were Ag 3d, W 4f, and O 1s for all the samples. Additionally, for the doped samples, peaks related to Cu 2p and Zn 2p elements were identified, indicating that the samples were pure with no impurities detected. The C 1s contamination peak can be ascribed to adventitious hydrocarbons from the XPS instrument.

**Figure 3:**
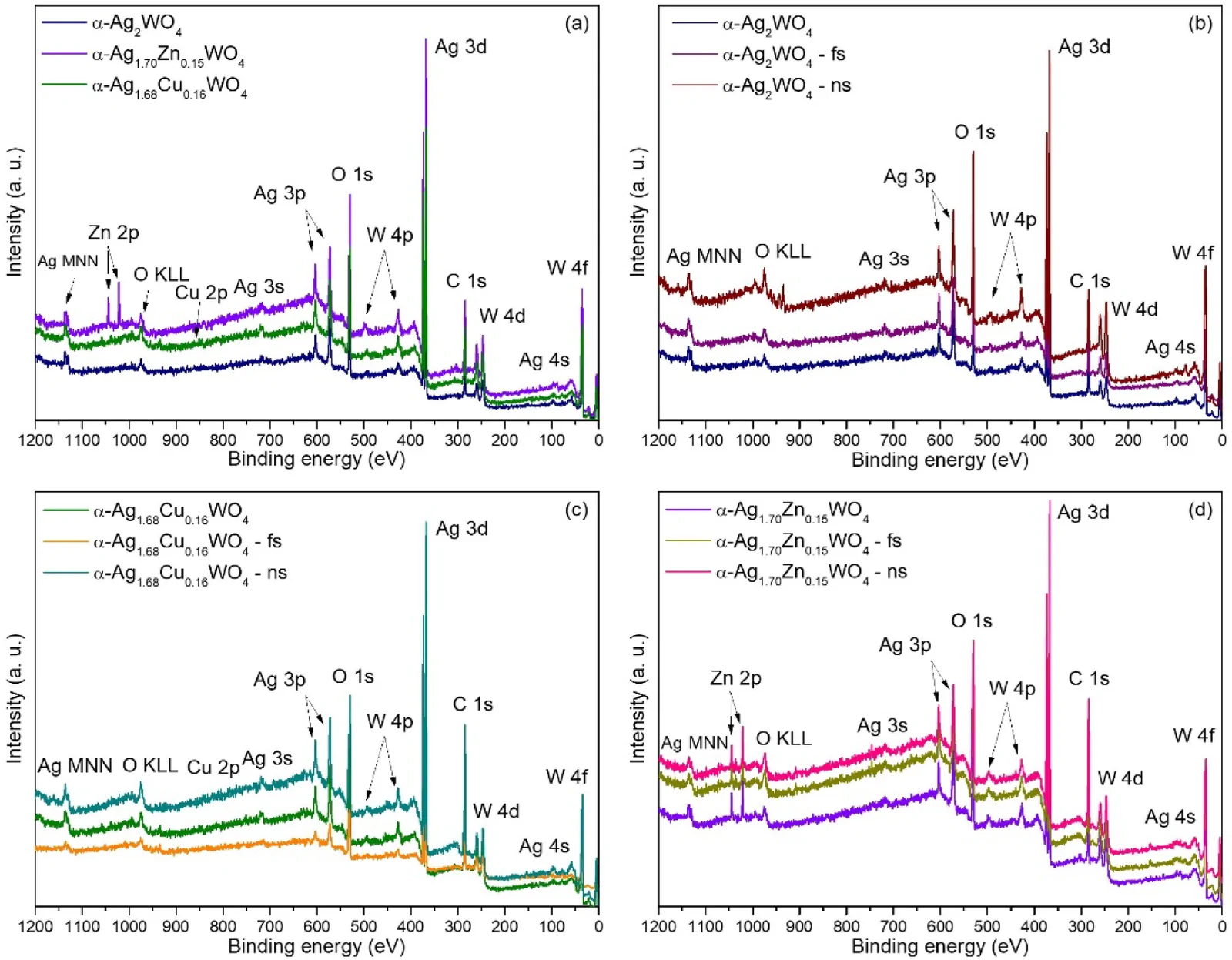
XPS survey spectrum of α-Ag_2–2*x*_M*_x_* WO_4_ (M = Cu^2+^ and Zn^2+^): (a) nonirradiated α-Ag_2_WO_4_, α-Ag_1.68_Cu_0.16_WO_4_, and α-Ag_1.70_Zn_0.15_WO_4_; (b) fs and ns laser-irradiated α-Ag_2_WO_4_; (c) fs and ns laser-irradiated α-Ag_1.68_Cu_0.16_WO_4_; and (d) fs and ns laser-irradiated α-Ag_1.70_Zn_0.15_WO_4_ samples.

A high-resolution spectrum of each element was carried out for further analysis of the materials. The high-resolution Ag 3d spectra in the range of 364–378 eV present two peaks, as shown in Figure ESI-1­(a–i). Two deconvolution components located at 367.4 and 373.4 eV (Δ = 6 eV) are due to Ag 3d_5/2_ and Ag 3d_3/2_ indicating the presence of Ag^+^ ions. Another two deconvolution components presented at 368.2 and 374.2 eV (Δ = 6 eV) are due to the presence of Ag^0^ which can be related to the surface-coated Ag nanoparticles on the nonirradiated materials.^55,56^ For the fs- and ns-irradiated materials, the Ag^0^ components can also be ascribed to the metallic Ag growth both on the surface and in the bulk of the materials.

The high-resolution W 4f spectra in the range of 31–44 eV present two peaks, as shown in Figure ESI-2­(a–i). Two components located at 34.8 and 37.0 eV (Δ = 2.2 eV) are due to the W 4f_7/2_ and W 4f_5/2_ which confirm the W^6+^ oxidation state. Another two components can be ascribed to W^5+^ at 34.2 eV (W 4f_7/2_) and 36.4 eV (W 4f_5/2_) (Δ = 2.2 eV), and one component at ∼41 eV can be assigned to W 5p_3/2_.^29,55,57^ These components are associated with W clusters, which were little affected by the modifications caused by laser irradiation.

The high-resolution O 1s spectra in the range of 527–536 eV are presented in Figure ESI-3­(a–i). In general, the peaks located at 529.6, 531.2, and 532.8 eV correspond to the Ag–O bond, W–O, and OH group of the surface water molecule. The broad peak overlapping in the region of ∼530 eV can be ascribed to the crystal lattice oxygen of the microcrystals.^58–63^ It was observed that similar component areas were present for the nonirradiated samples. However, the presence of any kind of dopant (Cu or Zn) combined with any kind of laser irradiation (ns or fs) promoted an increase in the area of the component related to the OH group of the surface water molecule, as observed in Figure ESI-4­(e, f, h, and i). This is a result of laser irradiation, which favors the reorganization of the Ag and W clusters, allowing more sites for OH adsorption on the surface of the materials.

The high-resolution Cu 2p spectra in the range of 926–965 eV are shown in Figure ESI-4­(a–c). The two component peaks at 934 and 954 eV (Δ = 20 eV) may be assigned to Cu 2p_3/2_ and Cu 2p_1/2_, respectively. Chemical state differentiation of copper can be difficult with XPS alone, but two Cu satellite peaks located at 942 and 962 eV (Δ = 20 eV) indicate the 2+ oxidation state of this element. The satellite peaks are associated with the lack of symmetry in the electronic configuration of d^9^ of Cu^2+^. Additionally, the Cu 2p_3/2_ peak in Cu­(II) is shifted and is much broader compared to Cu­(I).^64,65^

The high-resolution Zn 2p spectra in the range of 1015–1065 eV are shown in Figure ESI-5­(a–c). The Zn 2p peak has significantly split spin–orbit components (Δ = 23 eV), and these two component peaks are located at 934 and 954 eV (Δ = 23 eV), which can be assigned to Zn 2p_3/2_ and Zn 2p_1/2_, respectively.^66,67^

The XPS quantification of Cu^2+^ and Zn^2+^ elements was carried out as an estimate of these elements in the samples before irradiation. The atomic amounts calculated were 0.18% for Cu^2+^ and 0.80% for Zn^2+^. These values were close to the experimental values, and deviations occurred due to XPS analysis being a surface analysis, with bulk compositions not considered in the calculations.

### Raman Spectroscopy Analysis

Figure 4a–d shows the Raman spectra of the α-Ag_2_WO_4_, α-Ag_1.68_Cu_0.16_WO_4_, and α-Ag_1.70_Zn_0.15_WO_4_ samples nonirradiated and irradiated by ns and fs lasers, within the range 70–1000 cm^–1^.

**Figure 4:**
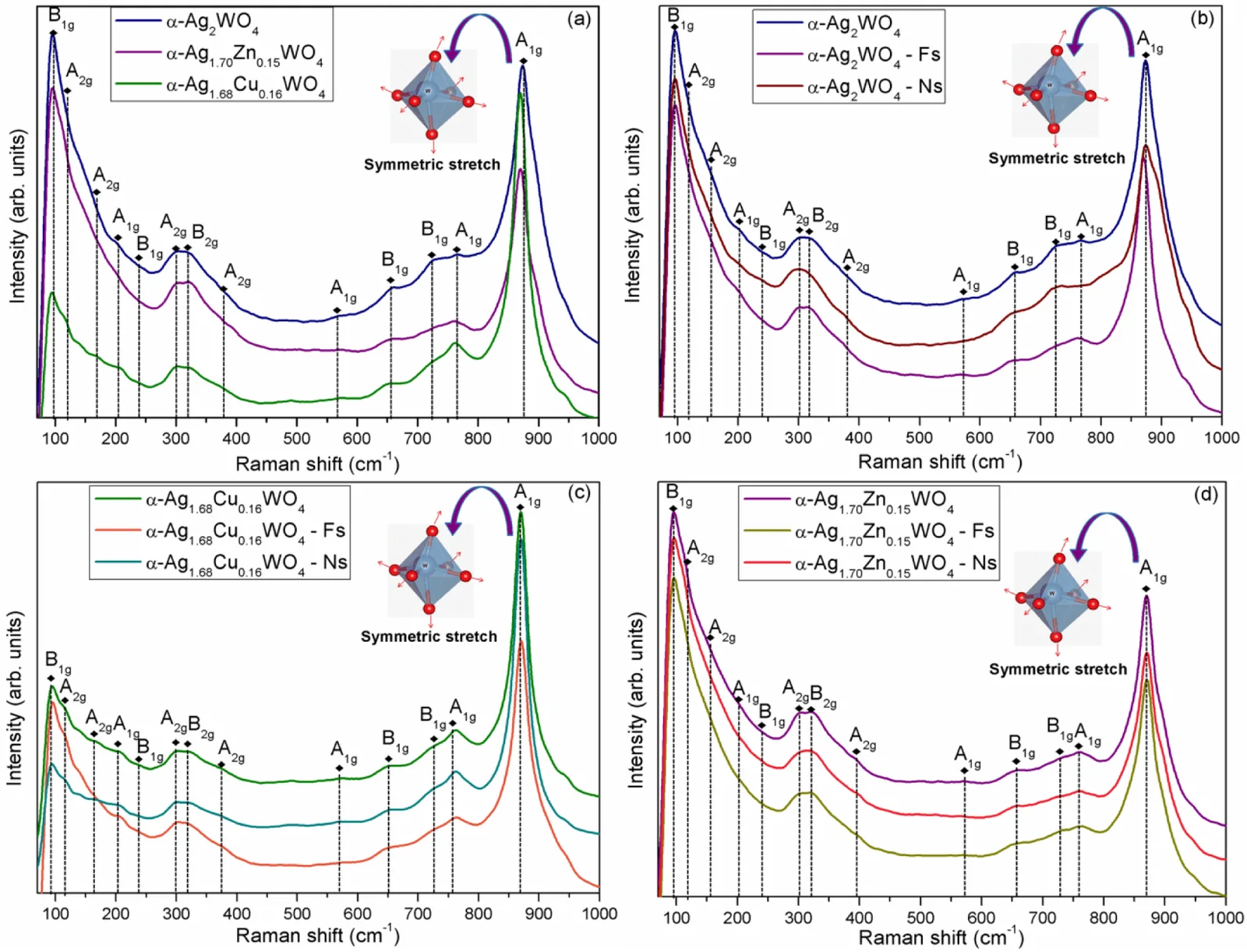
Raman-active modes of α-Ag_2–2*x*_M*_x_* WO_4_ (M = Cu^2+^ and Zn^2+^): (a) nonirradiated α-Ag_2_WO_4_, α-Ag_1.68_Cu_0.16_WO_4_, and α-Ag_1.70_Zn_0.15_WO_4_; (b) fs and ns laser-irradiated α-Ag_2_WO_4_; (c) fs and ns laser-irradiated α-Ag_1.68_Cu_0.16_WO_4_; and (d) fs and ns laser-irradiated α-Ag_1.70_Zn_0.15_WO_4_ samples.

An analysis of the results reveals that the Raman-active modes are characteristic of the orthorhombic structure. The vibrational active modes of α-Ag_2_WO_4_ compounds are classified into two groups: internal and external modes and/or lattice phonons, such as *WO*_4_ tetrahedral and Ag^+^, respectively.^7,19,49^ The active modes in the range from 1000 to 500 cm^–1^ are assigned to internal modes of [*WO*_6_] clusters, and the active modes in the range below 500 cm^–1^ are related to external vibrational modes of [*A_g_O*_*x*_] (where *x* = 2, 4, 6, and 7) clusters.^21,56^

Figure 4 shows the A_1g_, A_2g_, B_1g_, and B_2g_ vibrational modes, where the most intense peak at 876 cm^–1^ (A_1g_) is attributed to the symmetric stretching vibration of the O–W–O bond angle. The Raman active modes in the range 876–500 cm^–1^ are assigned to asymmetric and symmetric stretching vibration modes of the O–W–O bond angle, as well as interchain deformation and lattice modes.^56^

Figure 4a displays the Raman active modes of the α-Ag_2_WO_4_, α-Ag_1.70_Zn_0.15_WO_4_, and α-Ag_1.68_Cu_0.16_WO_4_ compounds. It can be observed that there is structural order at short range, maintaining the orthorhombic structure for all the compounds. However, a small change is clearly seen between the α-Ag_2–2*x*_M_*x*_WO_4_ (M = Cu^2+^ and Zn^2+^) doped compounds in relation to pure α-Ag_2_WO_4_. First, it is observed that there is a small shift in all peaks, which is more prominent in the most intense peak at 876 cm^–1^ (A_1g_) which peak becomes more intense and narrower with doping, indicating a more ordered structure at short range. However, for the active modes in the range 500–70 cm^–1^ attributed to vibrational modes of [*A_g_O*_*x*_] (where *x* = 2, 4, 6, and 7) clusters, a pronounced change in the peaks is observed. For instance, some peaks are not observed in the α-Ag_2–2*x*_M_*x*_WO_4_ (M = Cu^2+^ and Zn^2+^) doped samples. In the range 250–70 cm^–1^ it is seen a more defined set of peaks of α-Ag_1.68_Cu_0.16_WO_4_ compound, which is different from the sample doped with the Zn^2+^ cation. Another important characteristic is observed in the range of 96–500 cm^–1^, where a decrease in the peak intensity occurs for the doped samples. This result is indicative of a successful doping process that induced the formation of different structural defects, caused by changes in bonding length and angle along the [O–Ag–O]–[O–W–O]–[O–M–O] (M = Zn^2+^ and Cu^2+^) network.^7^

Figure 4b–d shows the Raman active modes of α-Ag_2_WO_4_, α-Ag_1.68_Cu_0.16_WO_4_, and α-Ag_1.70_Zn_0.15_WO_4_ compounds irradiated by fs and ns lasers. It is clearly observed that the active modes of the samples irradiated by fs and ns lasers are much broader in relation to the nonirradiated samples. Also, a small shift is observed for the modes, and some active modes are not seen, mainly for the α-Ag_2_WO_4_ and α-Ag_2–2*x*_M_*x*_WO_4_ (M = Zn^2+^and Cu^2+^) samples irradiated by ns lasers. This behavior is mainly observed below 500 cm^–1^ that is characteristic of external vibrational modes of [*A_g_O*_*x*_] (where *x* = 2, 4, 6, and 7) clusters. Moreover, according to Lin et al.,^56^ the asymmetry and broadening of the peak at 870 cm^–1^ (A_1g_) are attributed to the symmetric stretching of O–W–O moieties, indicating that there may be a different degree of distortion in the [*WO*_6_] clusters for the samples irradiated by fs and ns lasers.

### FT-IR Spectroscopy Analysis

FTIR analyses were used to elucidate the [*WO*_6_] cluster distortion caused by the insertion of dopants and the fs/ns laser irradiation into the samples.

Figure 5 illustrates FTIR spectra in the range from 50 to 1100 cm^–1^, which are characteristic of the distorted tetrahedral $\left[WO_{4}^{2-}\right]$ moiety. In general, all the samples show intense peaks in the range of 778–845 cm^–1^ and 380–520 cm^–1^, which are attributed to asymmetric stretching vibration modes of the W–O–W and O–W–O, characteristic of tetragonal $\left[WO_{4}^{2-}\right]$ clusters, respectively. The active mode at 614 cm^–1^ is related to bridging oxygen atoms in the W_2_O_2_ asymmetric stretching, and the absorption bands at 659 and 704 cm^–1^ are associated with the vibrations of the O–W–O bond angle.^7,20,56^ In the range of 50–250 cm^–1^ the active modes are characteristic of the Ag–O bonds. The active modes between 50 and 88 cm^–1^ are attributed to rotational modes of the Ag–O–W; the mode at 127 cm^–1^ is assigned to the vibrational mode of the O–Ag–Ag bond angle; the mode at 160 cm^–1^ is characteristic of the O–Ag stretching bond; and the modes between 196 and 250 cm^–1^ are attributed to bending vibrational modes of the Ag–O–W and O–Ag–O bond angles.

**Figure 5:**
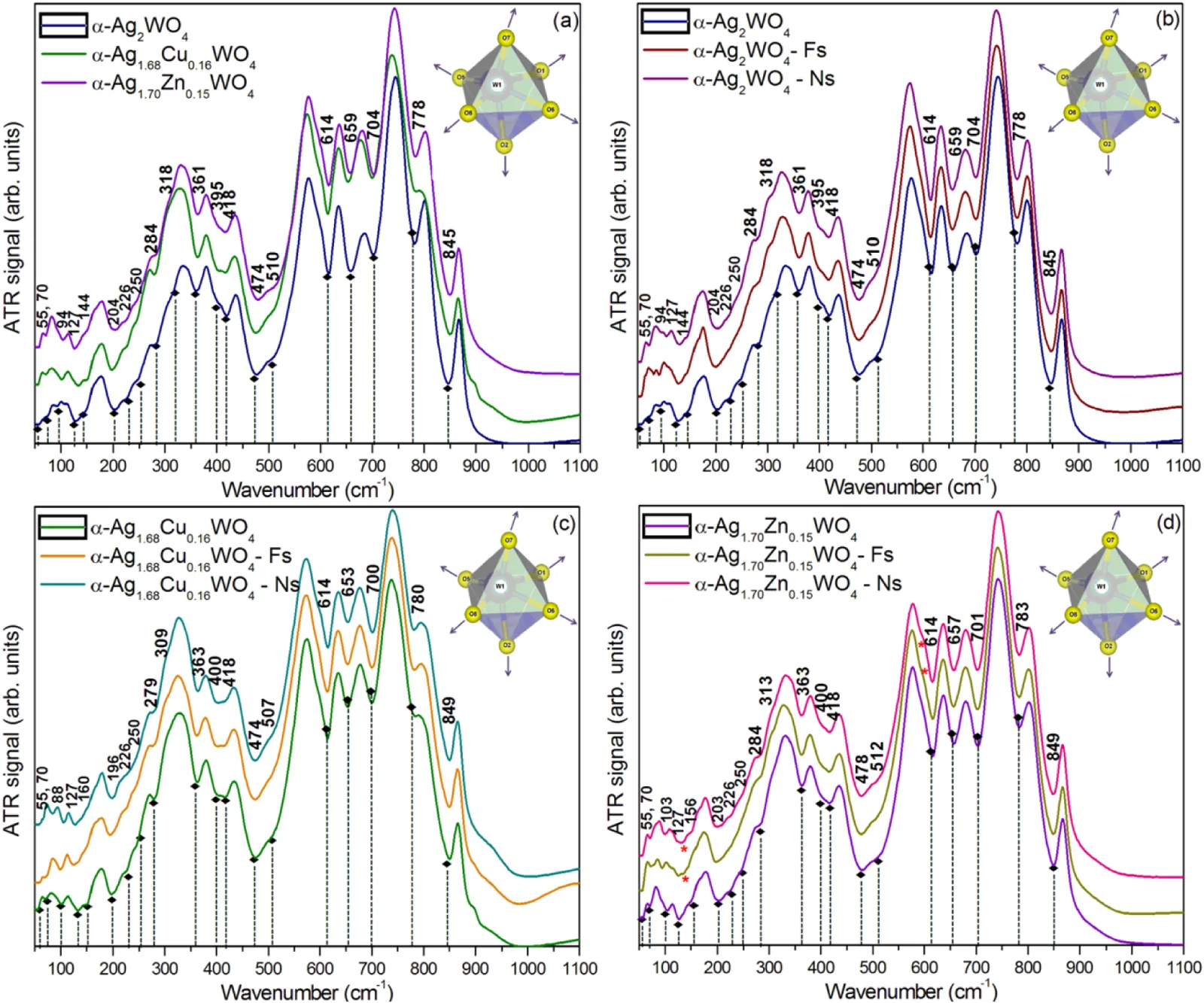
FTIR spectra of α-Ag_2–2*x*_M*_x_* WO_4_ (M = Cu^2+^ and Zn^2+^): (a) nonirradiated α-Ag_2_WO_4_, α-Ag_1.68_Cu_0.16_WO_4_, and α-Ag_1.70_Zn_0.15_WO_4_; (b) fs and ns laser-irradiated α-Ag_2_WO_4_; (c) fs and ns laser-irradiated α-Ag_1.68_Cu_0.16_WO_4_; and (d) fs and ns laser-irradiated α-Ag_1.70_Zn_0.15_WO_4_ samples.

Figure 5a shows a small shift of the modes to longer wavenumber of α-Ag_2–2*x*_M_*x*_WO_4_ (M = Cu^2+^ and Zn^2+^) samples compared α-Ag_2_WO_4_ sample and some active modes decrease in their intensities. This behavior is clearly observed at 778 cm^–1^, and in the regions of 650–704 cm^–1^, 450–510 cm^–1^, and 390–420 cm^–1^ which are attributed to the vibration modes of the W–O–W and O–W–O frameworks of the $\left[WO_{4}^{2-}\right]$ group. These findings prove the success of the Ag^+^ substitution by the Zn^2+^ and Cu^2+^ cations in the α-Ag_2–2*x*_M_*x*_WO_4_ (M = Zn^2+^ and Cu^2+^) samples,^7^ which causes changes in the O–W–O bond angles and Ag–O distances. Additionally, Figure 5b presents some shifting and a decrease in the intensity of absorption modes due to fs and ns irradiation of Ag_2_WO_4_ as expected, due to changes in the [AgO_*x*_] clusters, which also affect [WO_6_] clusters.

Figure 5c and d shows the FTIR spectra of the α-Ag_2–2*x*_M_*x*_WO_4_ (M = Zn^2+^ and Cu^2+^) samples irradiated by fs and ns lasers. The results indicate a decrease in the intensity of absorption modes, and some active modes disappear. This behavior is observed mainly in the range of 770–850 cm^–1^ and 55–130 cm^–1^ concerning to the asymmetric stretch characteristic of $\left[WO_{4}^{2-}\right]$ moiety and [*A_g_O*_*x*_] (where, *x* = 2, 4, 6 and 7) clusters, respectively. It can be concluded that the substitution of Ag^+^ by Cu^2+^/Zn^2+^ and fs/ns irradiation induces a larger degree of distortions in the O–W–O and O–Ag–O bond angles, and different degrees of structural order–disorder at short range in α-Ag_2_WO_4_ lattice can be sensed.^21^

Therefore, the degree of [*WO*_6_] cluster distortions in the α-Ag_2–2*x*_M_*x*_WO_4_ (M = Zn^2+^ and Cu^2+^) samples is very different^56^ and this behavior is similar to the samples irradiated by fs and ns lasers. Thus, changes in the O–W–O and, consequently, in the O–Ag–O bond angles and bond lengths at [AgO_*x*_]–[WO_6_]–[MO_*x*_] (M = Zn^2+^ and Cu^2+^, *x* = 2, 4, 6, and 7) cluster networks take place. Therefore, the fs and ns laser irradiation induces different degrees of structural order–disorder at long-, medium-, and short-range and, consequently, induces different defect densities in α-Ag_2–2*x*_M_*x*_WO_4_ (M = Zn^2+^ and Cu^2+^) lattice.^56^

### FE-SEM, TEM, and HR-TEM Morphological Analysis

These techniques provide information about morphological changes, sizes, and distributions of particles in the context of the doping effect and different laser irradiation.

Figure 6A, B, and C shows the FE-SEM images of α-Ag_2_WO_4_ and α-Ag_2–2*x*_M_*x*_WO_4_ (M = Zn^2+^ and Cu^2+^) samples without laser irradiation. In these figures, it is possible to verify the effect of the substitution of Ag^+^by Zn^2+^ or Cu^2+^ in the α-Ag_2_WO_4_ crystal lattice, which has already been discussed in our recently published papers.^7^ As already illustrated, the α-Ag_2_WO_4_ exhibits rod-like morphology with hexagonal faces, different sizes, and uniform distribution. As demonstrated in our work, it is possible to observe the change in morphology with the substitution of Zn^2+^ and Cu^2+^ions in the α-Ag_2_WO_4_ crystal lattice and thus confirming the success of the substitution of Ag^+^ by Cu^2+^/Zn^2+^ ions. As illustrated in Figure 6B and C, changes in morphology, size, and distribution of the particles are evident, such as the appearance of some small and deformed rod-like structures with curved surfaces.^7^

**Figure 6:**
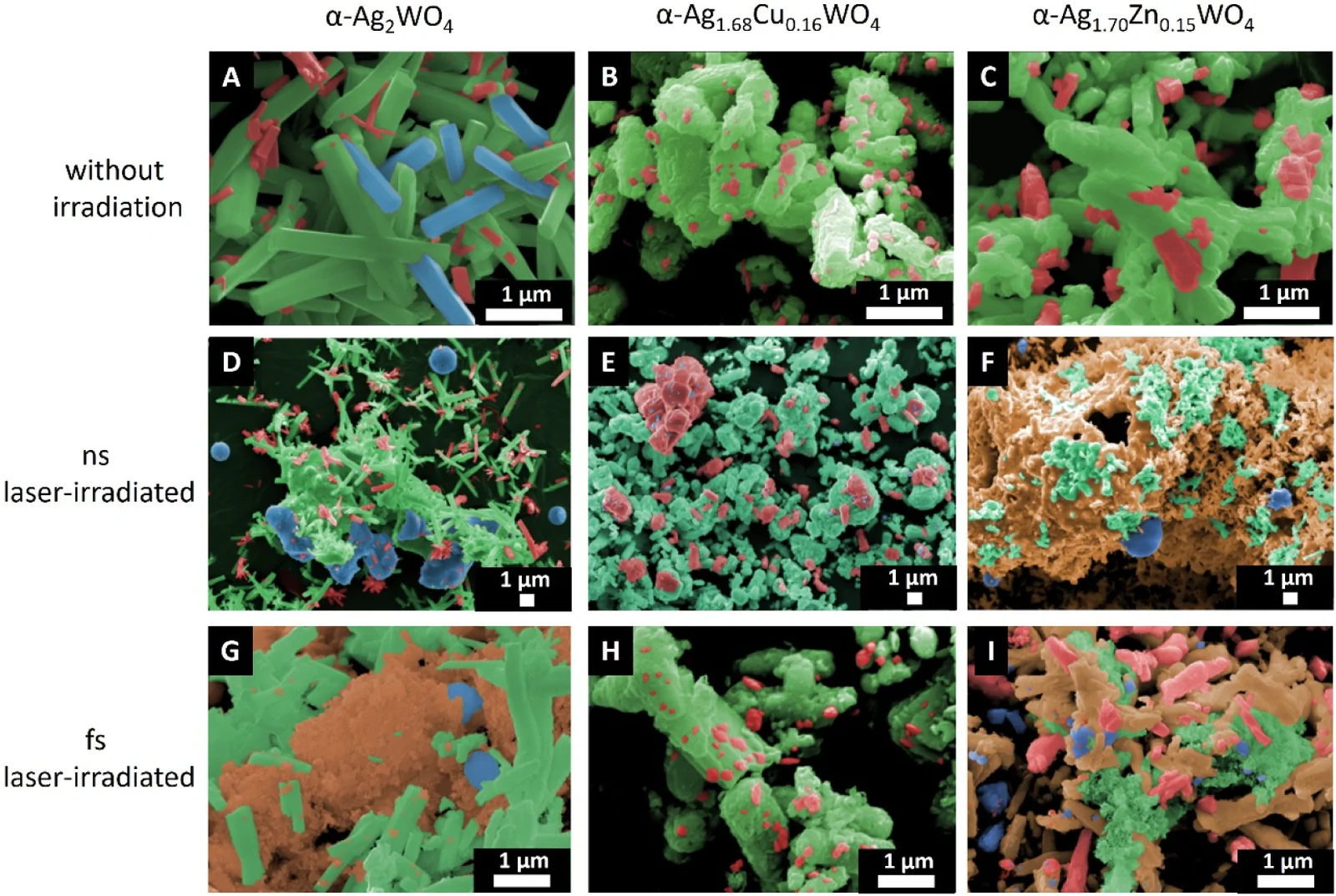
FE-SEM images of (A, B, C) α-Ag_2_WO_4_, α-Ag_1.68_Cu_0.16_WO_4_, and α-Ag_1.70_Zn_0.15_WO_4_ without irradiation; (D, E, F) α-Ag_2_WO_4_, α-Ag_1.68_Cu_0.16_WO_4_, and α-Ag_1.70_Zn_0.15_WO_4_ ns laser-irradiated; and (G, H, I) α-Ag_2_WO_4_, α-Ag_1.68_Cu_0.16_WO_4_, and α-Ag_1.70_Zn_0.15_WO_4_ fs laser-irradiated, respectively.

Figure 6D–I shows the α-Ag_2_WO_4_ and α-Ag_2–2*x*_M_*x*_WO_4_ (M = Zn^2+^ and Cu^2+^) samples irradiated by ns and fs lasers. The influence of ns and fs laser irradiation can be clearly observed. Figure 6D,E,F shows the samples irradiated by the ns laser, and in these figures, different morphologies can be observed, such as rod-like structures of various sizes, spherical particles, plates, and spherical nanoparticles. These morphologies probably occur due to the interactions of the laser with the samples, thus leading to the melting of the rods and, consequently, forming the plate and sphere morphologies. The morphological characteristics of these spherical nanostructures and the surface morphologies of α-Ag_2_WO_4_ and α-Ag_2–2*x*_M_*x*_WO_4_ (M = Zn^2+^ and Cu^2+^) formed through the irradiation were better defined by HR-TEM, which also confirmed their orthorhombic structure and the formation of metal elements.

Figure 6G,H,I presents the results of the samples irradiated by the fs laser, and these samples exhibit the same morphologies recently published by our group. Assis et al.^48^ showed the formation of spherical nanoparticles of α-Ag_2_WO_4_ isolated and/or agglomerated as well as rods and nanorods generated by fs irradiation. Figure 6H and I show, respectively, the α-Ag_2–2*x*_M_*x*_WO_4_ (M = Zn^2+^ and Cu^2+^) samples irradiated by the fs laser, and in these samples, deformed rods of various sizes and nanorods produced by laser irradiation are observed, along with clouds of spherical nanoparticles surrounding the rods. The clouds of spherical nanostructures and the metal elements were characterized by HR-TEM.

Through the TEM analysis, it was possible to verify the effect of the ns and fs laser irradiation on the α-Ag_2–2*x*_M_*x*_WO_4_ (M = Cu^2+^ and Zn^2+^) samples. Figure ESI-6­(a–f) shows the results for all samples irradiated by ns laser irradiation. The distance between the crystallographic planes present in the irregular spherical NPs was 2.36 Å, which can be indexed to the interplanar distance of the (111) family of planes in Ag^0^ with the typical *fcc* structure, in agreement with the JCPDS database (PDF 04-0783). This result confirms the extrusion of Ag species from the lattice of the initial semiconductor by its interaction with the radiation, forming a composite with the approximate formula Ag/α-Ag_2–2*x*_M_*x*_WO_4_ (M = Cu^2+^ and Zn^2+^). Additionally, some nonstoichiometric and amorphous NPs can be found on the initial material, which could be associated with NPs formed at the plasma plume jointly with the Ag NPs and allowed to settle on the surface of the materials, as also observed elsewhere.^48^

Figure 7a–c shows the results obtained from TEM for all samples irradiated by the femtosecond laser source.

**Figure 7:**
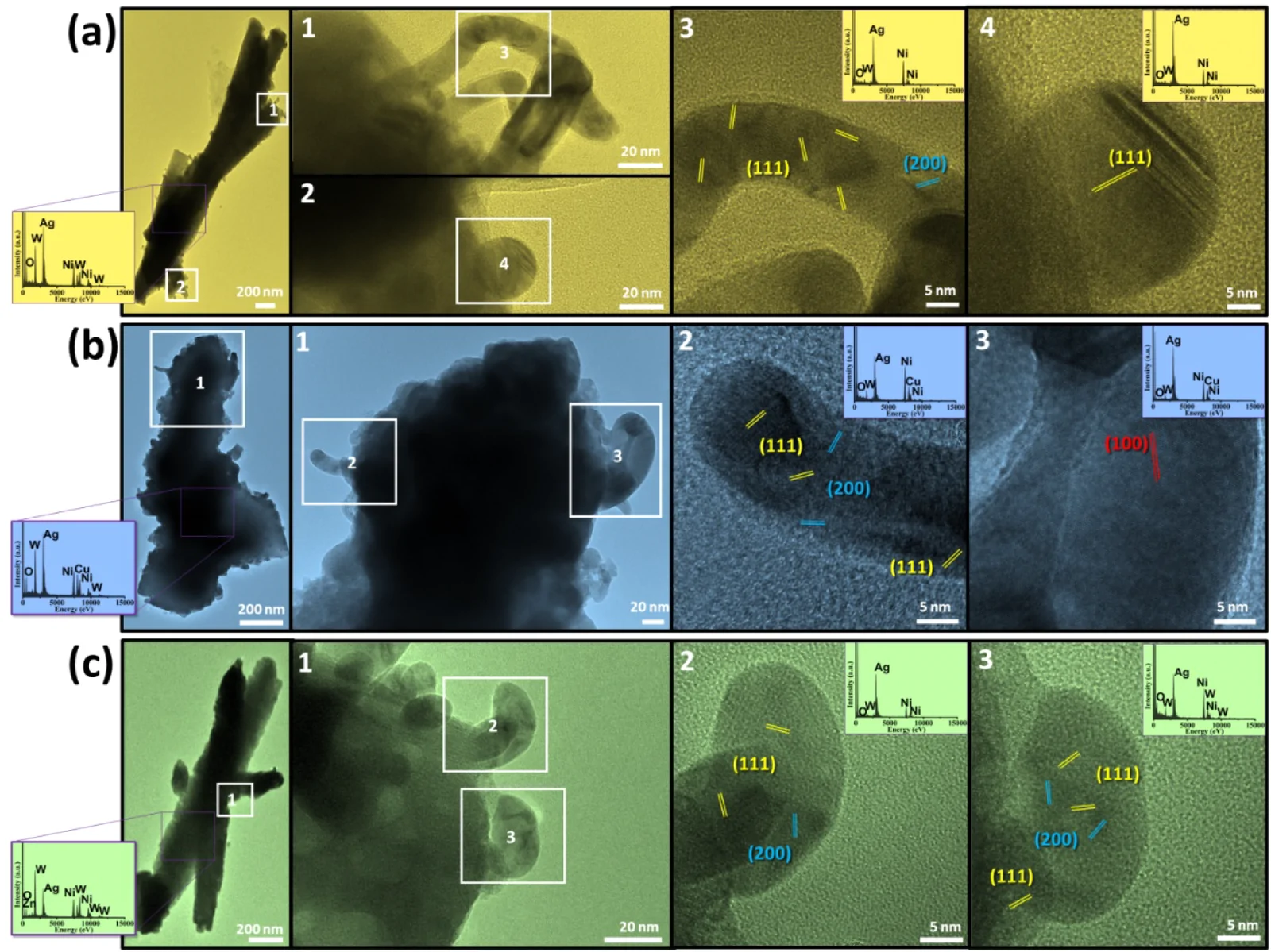
TEM and HR-TEM structural and morphological study of α-Ag_2–2*x*_M*_x_* WO_4_ (M = Cu^2+^ and Zn^2+^) irradiated by femtosecond laser: (a) α-Ag_2_WO_4_, (b) α-Ag_1.68_Cu_0.16_WO_4_, and (c) α-Ag_1.70_Zn_0.15_WO_4_.

As in the case of nanosecond laser irradiation, the morphology of the initial precursor microparticles remains unaltered after the laser processing, and the changes observed in each sample are only related to the Cu and Zn doping. The EDS characterization of the α-Ag_2–2*x*_M_*x*_WO_4_ microrods confirms the presence of only Ag, W, and O, as well as Cu and Zn in the doped samples (purple dotted squares). By analyzing the surface of the selected microrods, there is evident crystallization/deposition of several rod-like and spherical NPs (Regions 1 and 2). These NPs were further characterized by HR-TEM and EDS analysis (Regions 3 and 4), revealing a polycrystalline and monocrystalline nature for the nanorods and nanospheres, respectively. The distances between the crystallographic planes were 2.36 Å and 2.04 Å, which can be indexed to the interplanar distances of the (111) and (200) family of planes in Ag^0^ with the typical *fcc* structure (blue and yellow lines), as in the case of nanosecond laser irradiation. The EDS study demonstrates a major concentration of the Ag element in these NPs, and no significant concentration of other elements was observed, confirming the formation of Ag^0^ on the surface of α-Ag_2_WO_4_.

For the α-Ag_1.68_Cu_0.16_WO_4_ and α-Ag_1.70_Zn_0.15_WO_4_ samples in Figure 7b and c, similar trends compared to the pure α-Ag_2_WO_4_ sample were observed concerning the effects of femtosecond laser irradiation. The principal changes are the following: (i) inhibition of the formation/deposition of rod-like Ag NPs; (ii) higher concentration of Ag NPs with near-spherical morphologies compared to the pure Ag_2_WO_4_ irradiated sample (regions 1, 3, and 3 in Figure 7b and c), and (iii) the presence of the hexagonal polymorph (hcp) for Ag^0^. As observed in the HR-TEM micrograph of region 3 in Figure 7b, the measured distances of 2.50 Å for the crystallographic planes (red lines) match well with the (100) family of planes for this unusual polymorph of Ag^0^, in accordance with the JCPDS database (PDF41-1402). As demonstrated by our group, the fcc and hcp structures of Ag NPs could coexist when pure or doped α-Ag_2_WO_4_ samples are irradiated by an fs laser. Other analyzed regions for the three samples irradiated by the femtosecond laser are available in Figures ESI-7, ESI-8, and ESI-9. These analyses also render the identification of several mono- and polycrystalline Ag^0^ NPs, mainly with near-spherical morphologies. In these images, some amorphous material is observed embedded with the Ag NPs on the surface of the oxide microrods. The femtosecond laser irradiation causes the formation of a cloud composed of hot electrons, ions, and some other detached species labeled as the plasma plume. Some of these elements are deposited on the surface of our samples, forming a mixture of Ag NPs with amorphous and nonstoichiometric compounds mainly comprising Ag, W, O, and the Cu/Zn dopants.^48^

### Antibacterial Activity

Table 1 presents the MBC values of the synthesized materials against MRSA. The concentrations for the antimicrobial assays against MRSA were selected based on established protocols in the literature, following the Clinical and Laboratory Standards Institute (CLSI) guidelines, with a serial dilution range from 1.95 to 1000 μg/mL to accurately determine the MIC and MBC. The results demonstrate that all materials exhibited antibacterial activity, with significant variations in efficacy depending on composition and surface modification. α-Ag_2_WO_4_ showed an MBC of 125 μg/mL, while its modified versions exhibited a substantial improvement in antibacterial activity, with MBC values reduced to 31.25 μg/mL (ns) and 3.91 μg/mL (fs). This significant reduction indicates that the fs modification was the most effective in enhancing the antimicrobial properties of this material. MBC data were statistically analyzed using the nonparametric Kruskal–Wallis test, followed by Dunn’s multiple comparison post hoc test (*p* < 0.05).

**Table 1:** Minimum Bactericidal Concentrations (μg/mL) of Pristine and fs/ns Laser-Irradiated α-Ag_2_WO_4_, α-Ag_1.68_Cu_0.16_WO_4_, and α-Ag_1.70_Zn_0.15_WO_4_ against MRSA^a^

Material	MBC (μg/mL)
α-Ag_2_WO_4_	125^b^
α-Ag_2_WO_4_ ns	31.25^c^
α-Ag_2_WO_4_ fs	3.91^d^
α-Ag_1.68_Cu_0.16_WO_4_	31.25^c^
α-Ag_1.68_Cu_0.16_WO_4_ ns	15.63^c,d^
α-Ag_1.68_Cu_0.16_WO_4_ fs	15.63^c,d^
α-Ag_1.70_Zn_0.15_WO_4_	500^a^
α-Ag_1.70_Zn_0.15_WO_4_ ns	250^a,b^
α-Ag_1.70_Zn_0.15_WO_4_ fs	125^b^

a Different lowercase letters indicate statistically significant differences among materials (p &lt; 0.05).

The partial substitution of silver with copper in α-Ag_1.68_Cu_1.16_WO_4_ also resulted in increased antibacterial activity, with an MBC of 31.25 μg/mL in its unmodified form. However, ns and fs modifications only reduced the MBC to 15.63 μg/mL, suggesting that, in this case, the effect of modification was less pronounced than in pure α-Ag_2_WO_4_.

In contrast, the substitution of silver with zinc (α-Ag_1.71_Zn_1.15_WO_4_) led to a significantly higher MBC (500 μg/mL), indicating considerably lower antibacterial activity compared to the other materials. The ns modification reduced the MBC to 250 μg/mL, while the fs modification had a more pronounced effect, lowering it to 125 μg/mL. Although modification improved the antibacterial activity of this material, its performance remained inferior to that of the silver-only or silver–copper compounds. The antimicrobial assays revealed that femtosecond laser irradiation significantly enhanced the biocidal potency of α-Ag_2_WO_4_, achieving a 32-fold reduction in the MBC compared to the nonirradiated material.

Figure 8 presents the bacterial growth data (mean ± SD) of MRSA when exposed to subinhibitory and inhibitory concentrations of the synthesized materials. It can be observed that α-Ag_2_WO_4_ fs was the most effective material, significantly reducing bacterial viability even at low concentrations. In contrast, α-Ag_1.71_Zn_1.15_WO_4_ exhibited the weakest antibacterial effect, maintaining higher bacterial counts across all tested concentrations.

**Figure 8:**
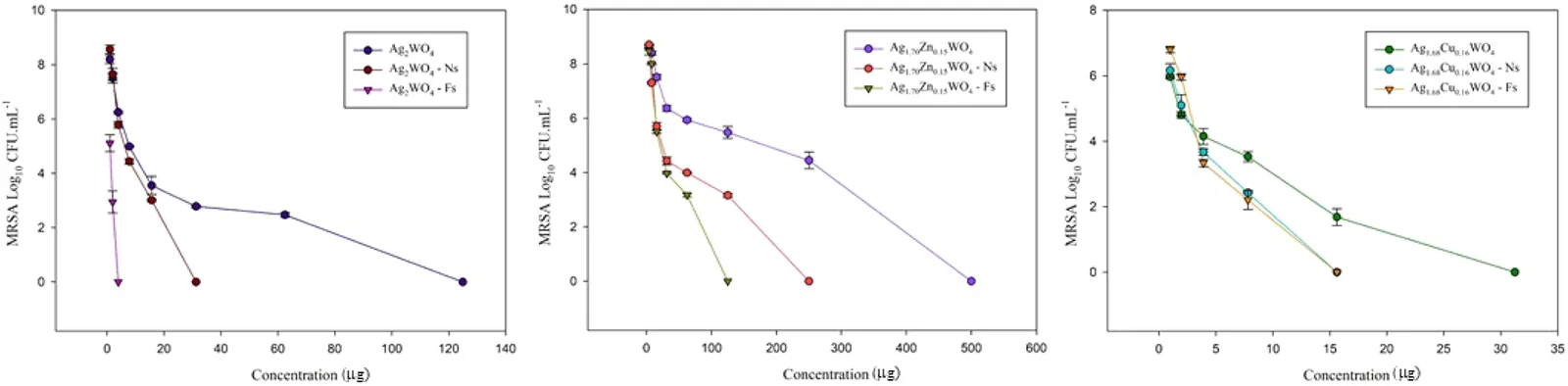
Bacterial growth of MRSA under subinhibitory concentrations of pristine and fs/ns laser-irradiated α-Ag_2_WO_4_, α-Ag_1.68_Cu_0.16_WO_4_, and α-Ag_1.70_Zn_0.15_WO_4_.

These findings suggest that the presence of silver is a key determinant of antibacterial activity, and that partial substitution with copper better preserves this property compared to substitution with zinc. Furthermore, fs modification proved to be the most effective in reducing MBC values, possibly due to an increase in surface area and enhanced material reactivity. The enhanced antimicrobial activity observed after laser irradiation (both ns and fs) may be associated with the generation of structural defects and surface modifications on the α-Ag_2–2*x*_M_*x*_WO_4_ microcrystals. Laser processing, particularly in the fs regime, promotes a rapid melting and solidification process, which may favor the formation of oxygen vacancies and surface disorders. These defects may act as active sites for the adsorption of water and oxygen molecules, potentially facilitating ROS generation, such as hydroxyl radicals (•OH) and superoxide anions (•O_2_^–^).^50,68,69^ Additionally, the laser-induced formation of Ag^0^ nanoparticles on the crystal surface, as confirmed by XPS analysis, may contribute synergistically to biocidal action, where the presence of metallic silver species and potential Ag^+^ release complement the defect-mediated ROS production. Furthermore, the incorporation of Zn^2+^ and Cu^2+^ ions into the Ag_2_WO_4_ lattice, combined with laser irradiation, may influence the electronic structure by creating intermediate energy levels within the bandgap. This may reduce electron–hole recombination (e^–^ – h^+^), potentially allowing more charge carriers to migrate to the surface and participate in redox reactions. These ROS induce oxidative stress, leading to lipid peroxidation of the bacterial cell membrane, protein denaturation, and ultimately, cell death, which is especially effective against the MRSA strain due to the potent synergistic effect of doping and ultrafast laser treatment.

Methicillin-resistant *Staphylococcos aureus* is widely found in cases of nosocomial infection, and because it is resistant to known treatments, it increases the morbidity and mortality of hospitalized patients. Thus, the development of new antibacterial agents is extremely important as an alternative to the commonly used ones, which are consequently not as efficient against the so-called “super bugs”.

## Conclusions

In summary, these results confirm the importance of introducing small amounts of substituents on α-Ag_2_WO_4_ during the synthesis and subsequent laser irradiation to significantly influence their properties and enhance the antimicrobial activity for important technological applications. XRD experimental results confirm the structural long-range order of the samples, while Raman and FT-IR spectroscopy analyses attested to their structural short-range order. XPS analysis confirmed the material’s surface purity as well as the oxidation states of the elements.

## Supplementary Material



## Notes

XPS core-level spectra of Ag, W, O, Cu, and Zn elements (PDF)
