# Supplementary material for: Femtosecond and Nanosecond Laser Irradiation to Enhance Antimicrobial Activity of α‑Ag 2–2x M x WO 4 (M x = Cu 0.15 and Zn 0.16 ) Microcrystals
Source: ACS Omega. 2026 Aug 18;11(34):51067–79. doi: 10.1021/acsomega.6c03200 (PMC13625093; doi:10.1021/acsomega.6c03200)
Supplement: Supplementary file 1 [file ao6c03200_si_001.pdf]

Electronic Supporting Information (ESI) for:

Femtosecond and nanosecond laser irradiation to enhance antimicrobial activity of α-$Ag_{2-2x}M_xWO_4$ (M_x = $Cu_{0.15}$ and $Zn_{0.16}$) microcrystals

Paula F. S. Pereira[1], *Camila C. de Foggi*[2], *Ivo M. Pinatti*[3], *Thales R. Machado*[4], *Henrique Alves*[5], *Cristina Paiva de Sousa*[5], *Carlos D. Buendía*[6], *Gladys Mínguez-Vega*[6], *Juan Andrés*[6] *and Elson Longo*[1]

[1]*CDMF, LIEC, Chemistry Department of the Federal University of São Carlos (UFSCar), P.O. Box 676, 13565-905 São Carlos, SP, Brazil*

[2]*Department of Conservative Dentistry, Faculty of Dentistry, Federal University of Rio Grande do Sul (UFRGS), 90035-003, Porto Alegre, RS, Brazil*

[3]*Department of Chemistry, Federal University of Maranhão (UFMA), Avenida dos Portugueses, 1966, São Luís 65080-805, MA, Brazil*

[4]*GNANO – Nanomedicine and Nanotoxicology Group, São Carlos, Institute of Physics, University of São Paulo, 13566-590 São Carlos, SP, Brazil.*

[5]*Department of Morphology and Pathology (DMP), Graduate Program of Biotechnology (PPGBiotec), Laboratory of Microbiology and Biomolecules (LaMiB)*

[6]*Department of Analytical and Physical Chemistry, University Jaume I (UJI), Castelló 12071, Spain*

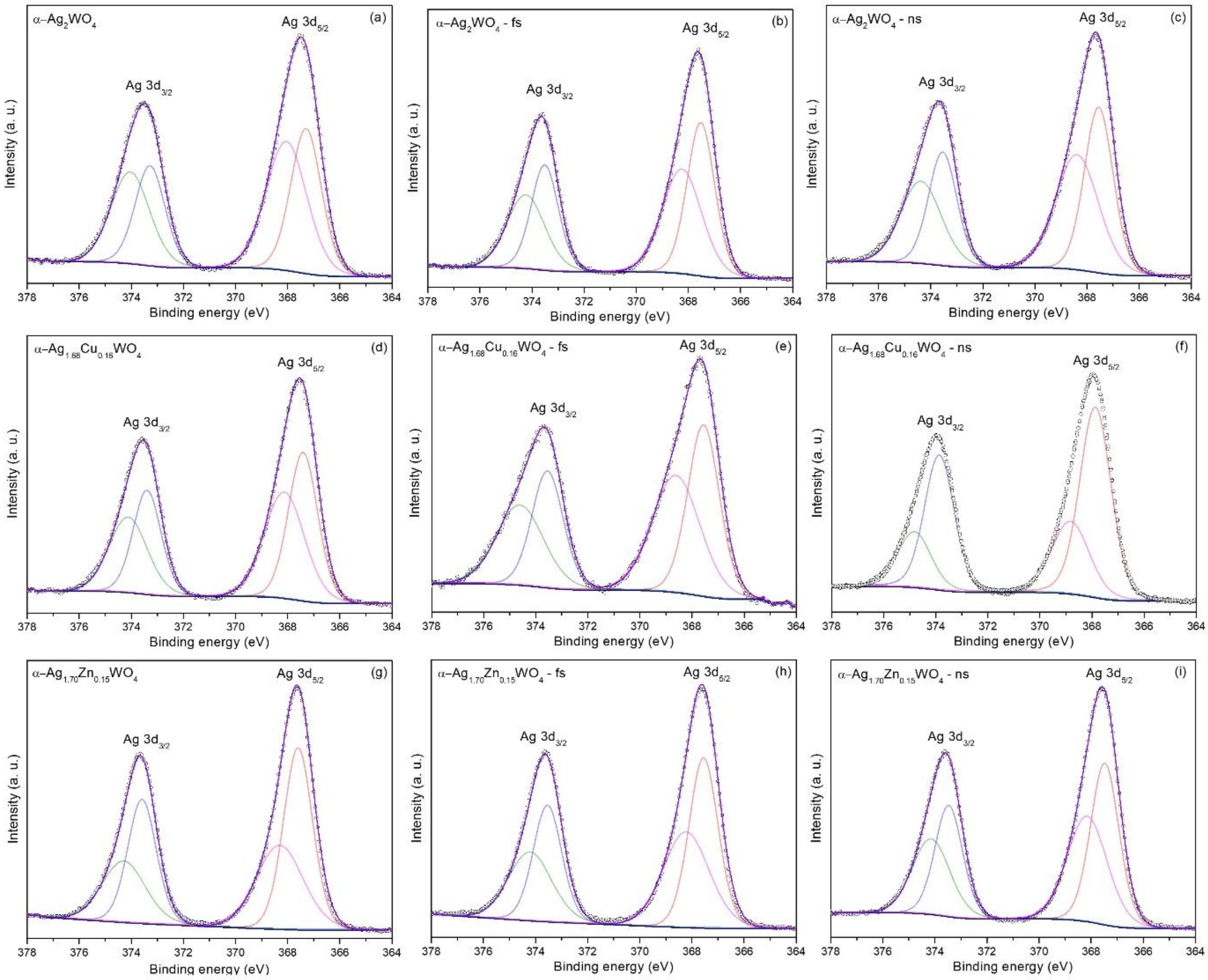

Fig. ESI-1 Core level spectra of Ag-3*d* of the α-$Ag_{2-2x}M_xO_4$ (M = Cu^{2+}, Zn^{2+}) microcrystals. (a), (d), (g) non-irradiated; (b), (e), (h) fs-irradiated; (c), (f), (i) ns-irradiated.

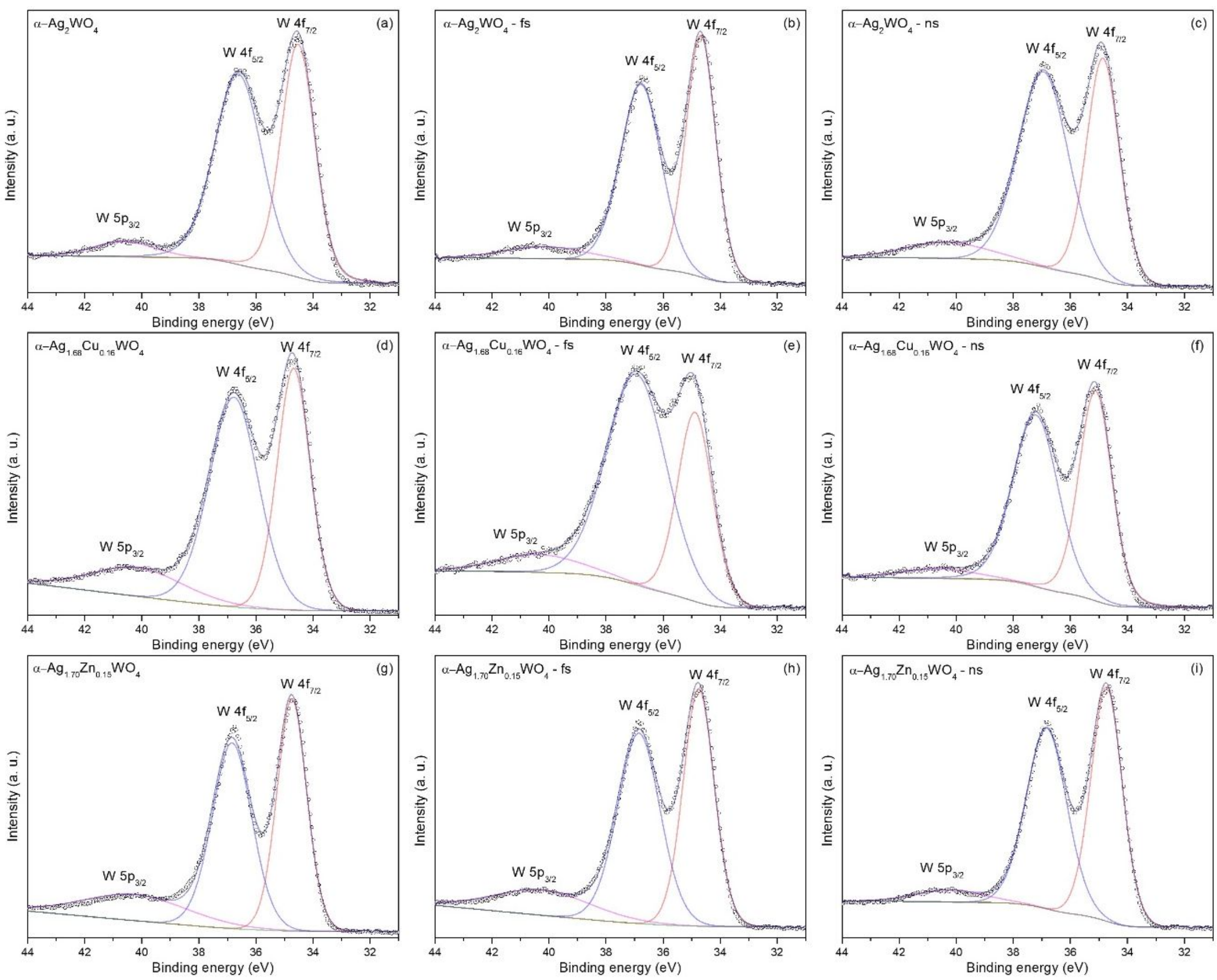

Fig. ESI-2 Core level spectrum of W-4*f* of the α-$Ag_{2\text{-}2x}M_xO_4$ (*M* = Cu^{2+}, Zn^{2+}) microcrystals. (a), (d), (g) non-irradiated; (b), (e), (h) fs-irradiated; (c), (f), (i) ns-irradiated.

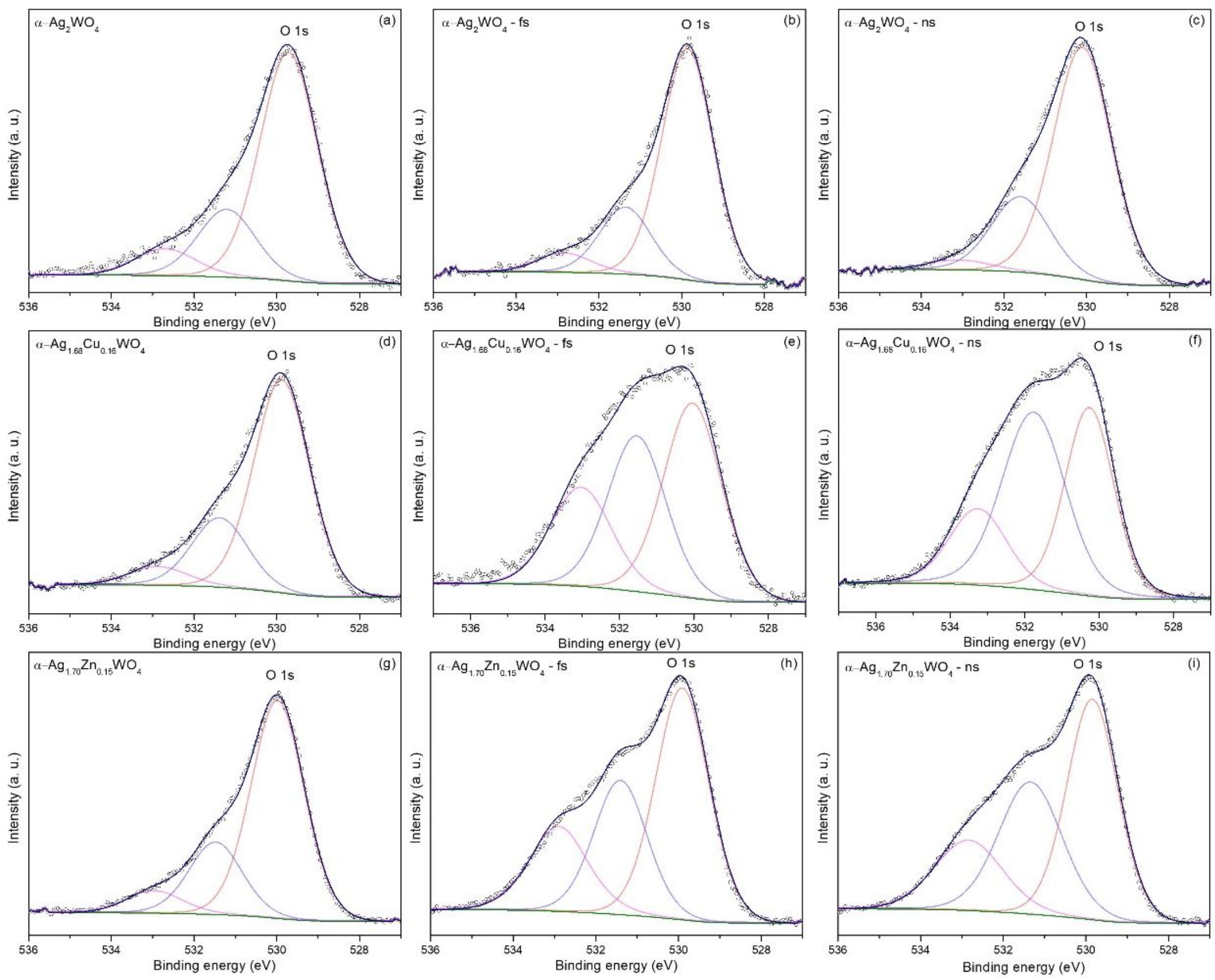

Fig. ESI-3 Core level spectrum of O-1*s* of the α-$Ag_{2-2x}M_xO_4$ (M = Cu^{2+}, Zn^{2+}) microcrystals. (a), (d), (g) non-irradiated; (b), (e), (h) fs-irradiated; (c), (f), (i) ns-irradiated.

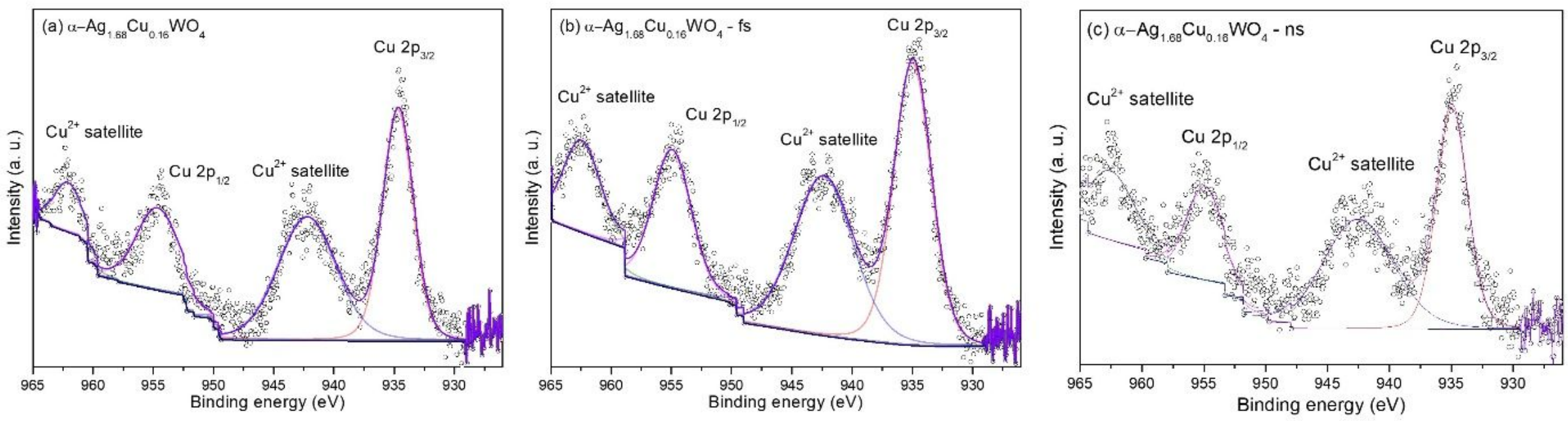

Fig. ESI-4 Core level spectrum of Cu-2*p* of the α-$Ag_{1.70}Cu_{0.16}O_4$ microcrystals. (a) non-irradiated, (b) fs, and (c) ns laser irradiated.

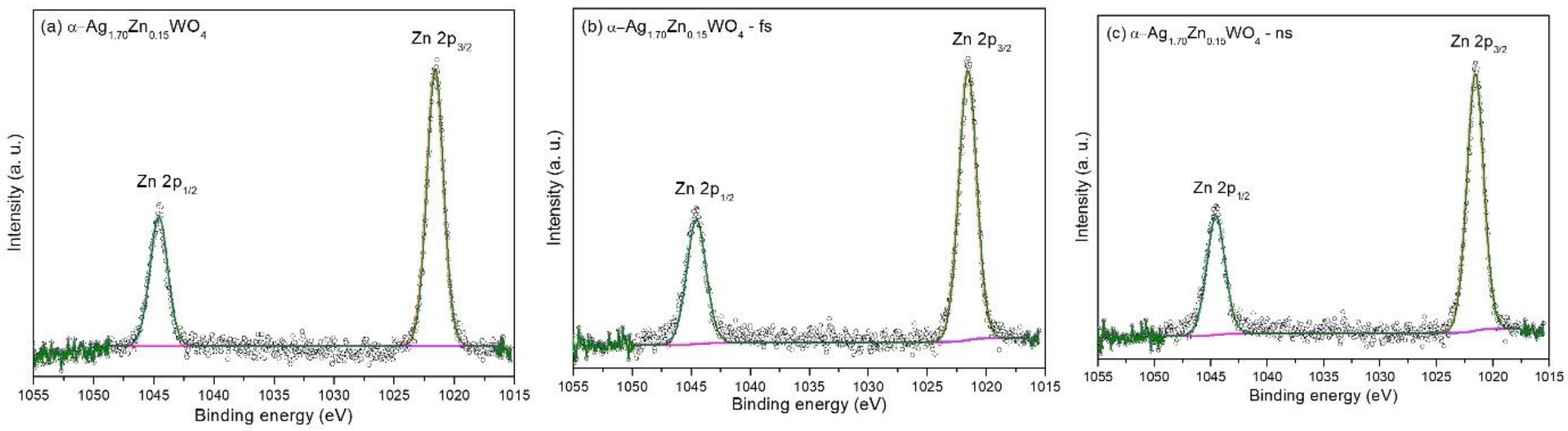

Fig. ESI-5 Core level spectrum of Zn-2*p* of the α-$Ag_{1.70}Zn_{0.15}O_4$ microcrystals. (a) non-irradiated, (b) fs-irradiated, and (c) ns-irradiated.

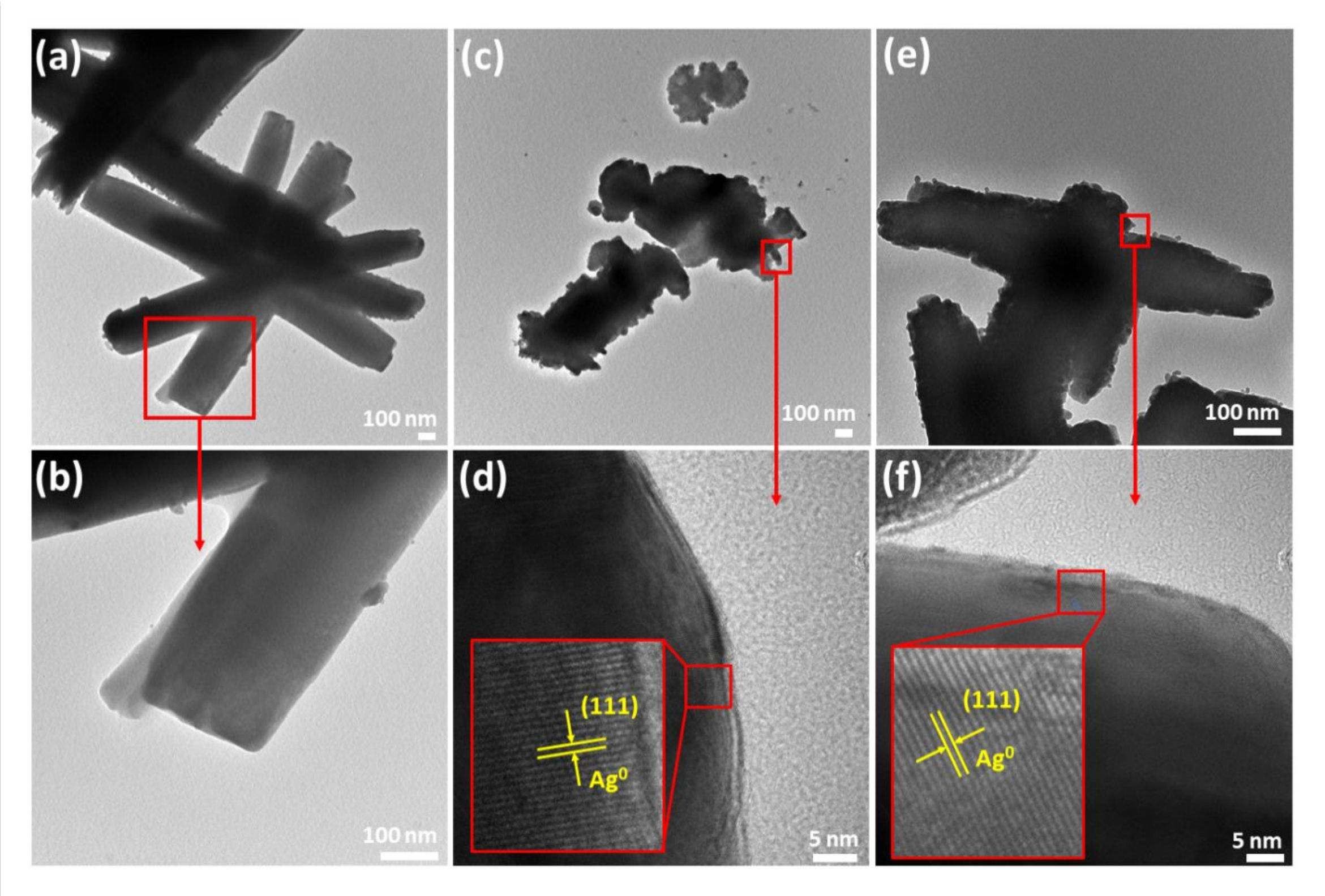

Fig. ESI-6 Structural and morphological TEM study of the α-Ag_2WO_4 pure and Cu/Zn doped α-Ag_2WO_4 samples treated by nanosecond laser irradiation. (a,b) α-Ag_2WO_4, (c,d) α-$Ag_{1.68}Cu_{0.16}WO_4$ and (e,f) α-$Ag_{1.70}Zn_{0.15}WO_4$.

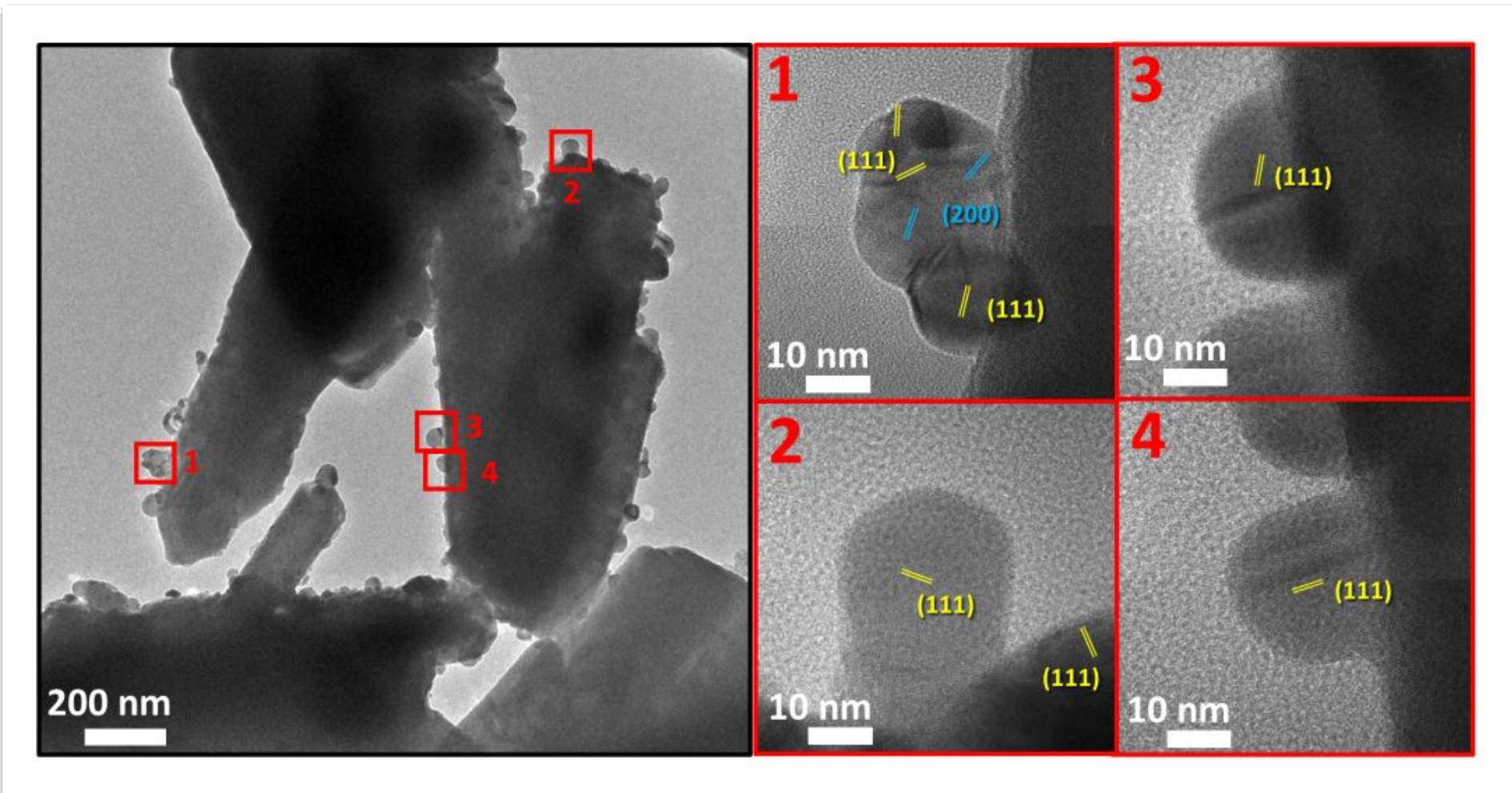

Fig. ESI-7 Structural and morphological study by TEM of the α-Ag_2WO_4 sample treated by femtosecond laser irradiation.

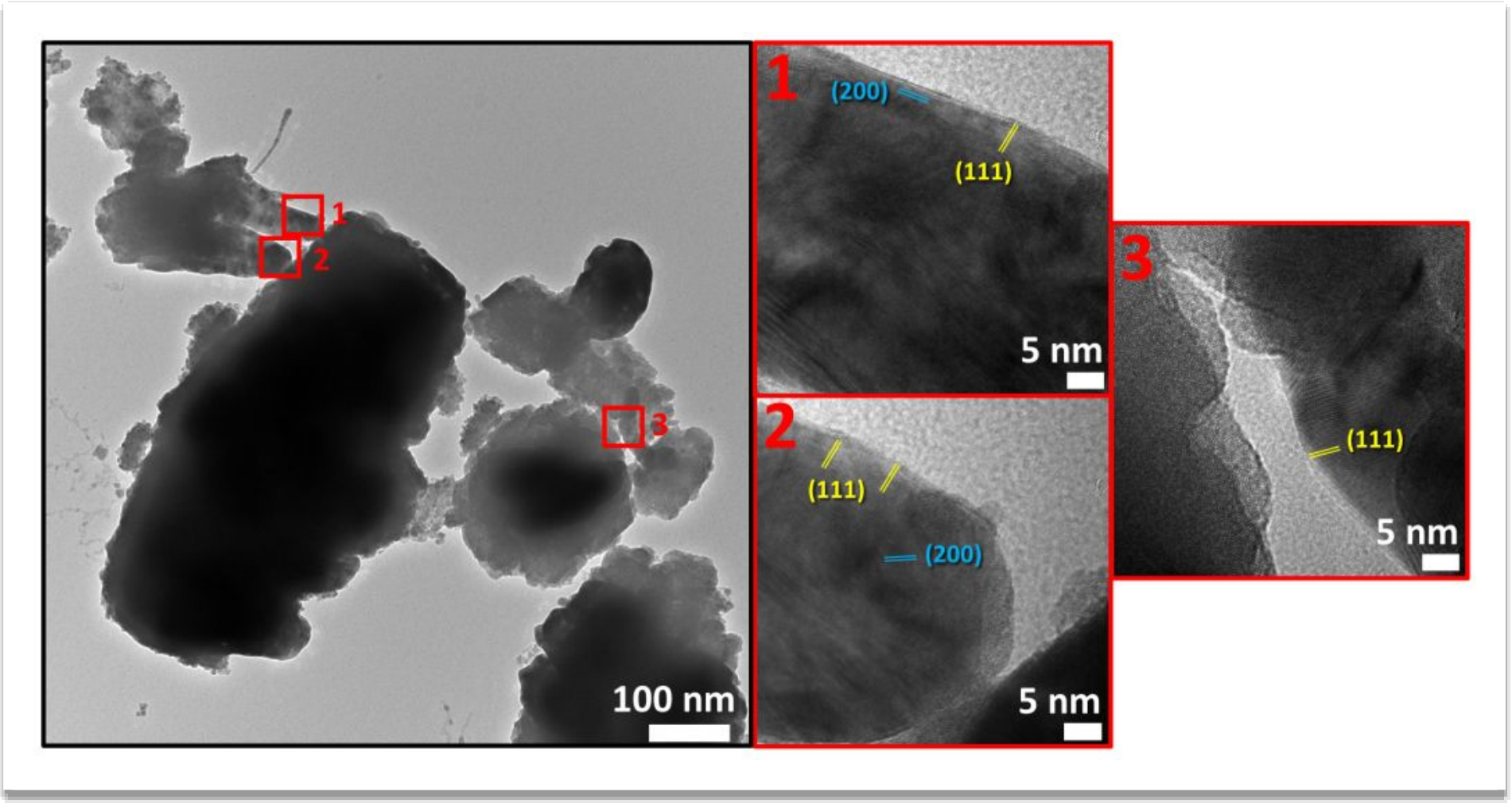

Fig. ESI-8 Structural and morphological study by TEM of the α-$Ag_{1.68}Cu_{0.16}WO_4$ sample treated by femtosecond laser irradiation.

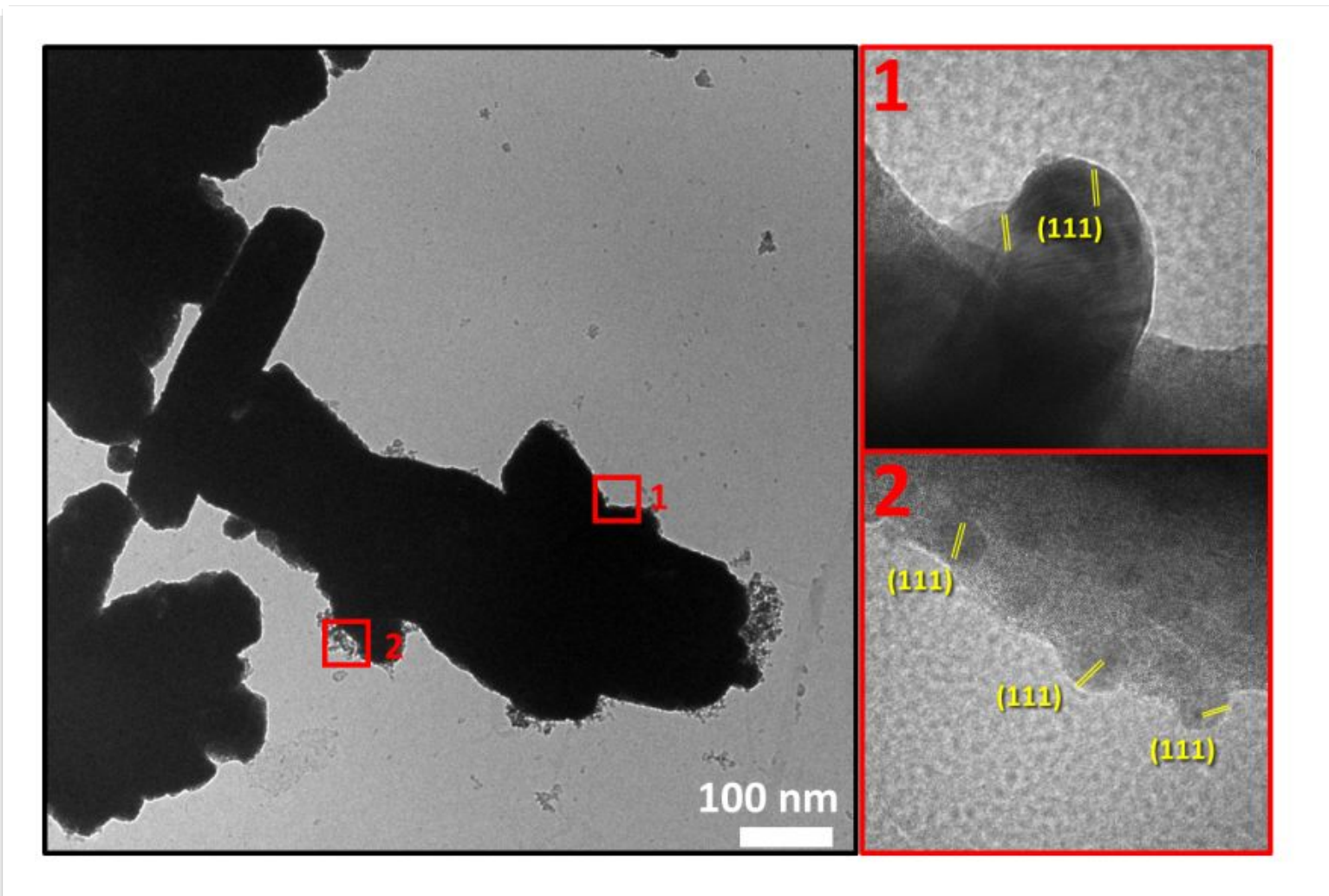

Fig. ESI-9 Structural and morphological study by TEM of the α-$Ag_{1.70}Zn_{0.15}WO_4$ sample treated by femtosecond laser irradiation.
